# Fatty Acid Synthase Promotes Hepatocellular Carcinoma Growth via S-Phase Kinase-Associated Protein 2/p27^KIP1^ Regulation

**DOI:** 10.3390/medicina60071160

**Published:** 2024-07-18

**Authors:** Antonio Cigliano, Maria M. Simile, Gianpaolo Vidili, Giovanni M. Pes, Maria P. Dore, Francesco Urigo, Eleonora Cossu, Li Che, Claudio Feo, Sara M. Steinmann, Silvia Ribback, Rosa M. Pascale, Matthias Evert, Xin Chen, Diego F. Calvisi

**Affiliations:** 1 Department of Medicine, Surgery and Pharmacy, University of Sassari, 07100 Sassari, Italy; acigliano@uniss.it (A.C.); simile@uniss.it (M.M.S.); gianpaolovidili@uniss.it (G.V.); gmpes@uniss.it (G.M.P.); mpdore@uniss.it (M.P.D.); francescourig@gmail.com (F.U.); cffeo@uniss.it (C.F.); patsper@uniss.it (R.M.P.); 2 Institute of Pathology, University of Regensburg, 93053 Regensburg, Germany; eleonoracossu26@gmail.com (E.C.); sara.steinmann@klinik.uni-regensburg.de (S.M.S.); matthias.evert@klinik.uni-regensburg.de (M.E.); 3 Department of Bioengineering and Therapeutic Sciences and Liver Center, University of California, San Fracisco, CA 94143, USA; cheli0315@yahoo.com (L.C.); xinchen3@hawaii.edu (X.C.); 4 Institute of Pathology, University of Greifswald, 17489 Greifswald, Germany; silvia.ribback@uni-greifswald.de; 5 Cancer Biology Program, University of Hawaii Cancer Center, Honolulu, HI 96813, USA

**Keywords:** hepatocellular carcinoma, lipogenesis, FASN, SKP2, p27^KIP1^, mouse models

## Abstract

*Background and Objectives:* Aberrant upregulation of fatty acid synthase (FASN), catalyzing de novo synthesis of fatty acids, occurs in various tumor types, including human hepatocellular carcinoma (HCC). Although FASN oncogenic activity seems to reside in its pro-lipogenic function, cumulating evidence suggests that FASN’s tumor-supporting role might also be metabolic-independent. *Materials and Methods*: In the present study, we show that FASN inactivation by specific small interfering RNA (siRNA) promoted the downregulation of the S-phase kinase associated-protein kinase 2 (SKP2) and the consequent induction of p27^KIP1^ in HCC cell lines. *Results:* Expression levels of *FASN* and *SKP2* directly correlated in human HCC specimens and predicted a dismal outcome. In addition, forced overexpression of SKP2 rendered HCC cells resistant to the treatment with the FASN inhibitor C75. Furthermore, FASN deletion was paralleled by SKP2 downregulation and p27^KIP1^ induction in the AKT-driven HCC preclinical mouse model. Moreover, forced overexpression of an SKP2 dominant negative form or a p27^KIP1^ non-phosphorylatable (p27^KIP1-T187A^) construct completely abolished AKT-dependent hepatocarcinogenesis in vitro and in vivo. *Conclusions:* In conclusion, the present data indicate that SKP2 is a critical downstream effector of FASN and AKT-dependent hepatocarcinogenesis in liver cancer, envisaging the possibility of effectively targeting FASN-positive liver tumors with SKP2 inhibitors or p27^KIP1^ activators.

## 1. Introduction

Hepatocellular carcinoma (HCC) is the most frequent primary liver malignancy and the second deadliest tumor worldwide [1,2,3,4]. While surgical resection and liver transplantation represent efficient therapeutic approaches against early-stage HCC, the advanced, inoperable disease treatment options are of limited benefit for the patients in terms of overall survival [4,5,6,7]. Several drugs have been approved to treat advanced HCC, such as the multikinase inhibitors Sorafenib, Lenvatinib, Regorafenib, Ramucirumab, and Cabozantinib, often characterized by modest and temporary efficacy [4,5,6,7]. More recently, it has been demonstrated that the administration of atezolizumab (a monoclonal antibody against programmed death-ligand 1) combined with bevacizumab (a monoclonal antibody targeting vascular endothelial growth factor) resulted in better overall and progression-free survival than sorafenib [8,9], generating new optimism for the treatment of this lethal tumor. Thus, a better understanding of HCC molecular pathogenesis is imperative to identify novel targets leading to effective therapies against liver cancer.

Fatty acid synthase (FASN) is a multi-domain enzyme synthesizing fatty acids. Specifically, FASN catalyzes the conversion of acetyl CoA to malonyl CoA, which undergoes additional elongation to palmitate, a precursor of long-chain fatty acid synthesis. Unlike most adult organs, high levels of FASN characterize the developing embryo and cancer cells [10,11,12,13]. In human HCC, previous studies conducted by several groups, including ours, revealed the augmented and coordinated expression of the major enzymes responsible for de novo lipogenesis, including FASN, ATP citrate lyase (ACLY), acetyl-CoA carboxylase (ACC), and stearoyl-CoA desaturase (SCD) [14,15,16]. Significantly, the increased levels of pro-lipogenic proteins were inversely correlated with the length of patients’ survival in this aggressive disease [14,17,18,19]. Mechanistically, we discovered that the increased de novo lipogenesis is positively regulated by the AKT/mTORC1/S6/SREBP1 signaling pathway in HCC [15]. Subsequent experiments revealed that the silencing of FASN diminishes HCC cell growth and increases apoptosis in vitro, and ablation of *FASN* completely suppresses AKT and AKT/c-Met driven HCC formation in mice [15,20,21]. More recently, another group obtained similar results in an mTOR-driven mouse hepatocarcinogenesis model [22]. However, overexpression of FASN, either alone or in association with other oncogenes such as c-Met and mutant NRas, did not drive liver carcinogenesis in the mouse, assigning a tumor-supporter role rather than a bona fide oncogenic function to FASN [21]. These studies proved that FASN and its mediated lipogenesis are necessary for HCC growth in vivo.

Mounting evidence indicates that, besides de novo lipogenesis, the mechanisms whereby FASN fuels cancer proliferation and survival could be multiple. For instance, it has been shown that FASN promotes the growth of the HepG2 hepatoblastoma cell line by upregulating the levels of c-Myc and β-Catenin protooncogenes [23]. In addition, FASN suppression triggered the downregulation of cyclin A, B1, and D1 in hepatoma cell lines in a p38MAPK-dependent and p53-independent manner [24]. Furthermore, FASN enhanced the interaction between USP11 and eIF4B in diffuse large-B cell lymphoma cell lines, thus promoting unconstrained protein translation and growth [25]. Moreover, global gene profiling of mouse HCC lesions revealed that FASN regulates numerous downstream targets involved in tyrosine metabolism, pyruvate metabolism, and estrogen signaling, among many others, besides lipogenic networks [26]. Therefore, FASN might contribute to carcinogenesis through several mechanisms independent of fatty acid biosynthesis [19]. Recently, a possible link between FASN and the S-phase kinase associated-protein kinase 2 (SKP2) protooncogene has been reported. Specifically, a previous study found that FASN blockade induces the cell cycle arrest of tumor breast cancer cells by decreasing SKP2 levels [27]. In addition, the group of Krishnakumar S. identified SKP2 as one of the genes downregulated explicitly following either inhibition or silencing of FASN in retinoblastoma cell lines [28,29]. Furthermore, we found that SKP2 is one of the genes downregulated by the FASN inhibitor TVB3664 in in vitro and in vivo models of liver cancer [26].

SKP2 is a pivotal component of the E3 ubiquitin ligase that controls the turnover of p27^KIP1^ and other critical tumor suppressor genes by triggering their proteolysis [30,31,32,33,34]. In particular, SKP2-dependent downregulation of p27^KIP1^ is required for cell cycle progression [35]. In this cellular event, p27^KIP1^ binds to and prevents the activation of cyclin E-CDK2 and cyclin D-CDK4 complexes, thus controlling the cell cycle progression at the G1 phase [36]. Therefore, it is unsurprising that low levels of various tumor suppressors, particularly p27^KIP1^, are inversely correlated with the elevated expression of SKP2 across multiple tumor types, including HCC [33,34,37,38]. Furthermore, we have previously shown in mice that SKP2 overexpression alone is not sufficient to induce malignant liver transformation, but it cooperates with oncogenic NRAS (NRASV12) to induce tumor development [39]. Moreover, SKP2 significantly accelerates AKT-dependent hepatocarcinogenesis, further substantiating its pro-oncogenic role [39].

The present study analyzes the possible relationship between FASN and SKP2 in HCC. In particular, we sought to determine whether AKT and FASN pro-malignant activity relies on SKP2. Using human HCC cell lines and mouse models of hepatocarcinogenesis, we revealed that SKP2 is a FASN downstream effector in liver cancer driven by AKT. Thus, targeting SKP2 might be a potential therapeutic strategy for treating these aggressive tumors displaying activation of the AKT/FASN axis.

## 2. Materials and Methods

### 2.1. Constructs and Reagents

The pT3-EF1α, pT3-EF1α-HA-myr-AKT1, pT3-EF1α-HA-SKP2, pT3-EF1α-Cre, and pCMV/sleeping beauty transposase (SB) plasmids have been described previously [21,39]. The human *SKP2* dominant negative (SKP2dn) cDNA was kindly provided by Dr. Michele Pagano (Laura and Isaac Perlmutter NYU Cancer Center, New York, NY, USA). This plasmid, also known as (ΔF)SKP2, consists of an F-box-deleted SKP2 mutant, which inhibits the degradation of p27^KIP1^ and sustains the stability of p27^KIP1^ [30]. The human p27^KIP1-T187A^ cDNA was purchased from Addgene (#23048). The SKP2dn and p27^KIP1-T187A^ cDNAs were cloned into a pT3-EF5a-HA and pT3-EF5a-V5 vectors, respectively, via the Gateway polymerase chain reaction (PCR) cloning strategy (Invitrogen, Carlsbad, CA, USA). The plasmids encoding the gene(s) of interest, along with sleeping beauty transposase (pCMV/SB) in a ratio of 25:1, were diluted in 2 mL saline (0.9% NaCl) for each mouse. Plasmids were purified using the Endotoxin-free Maxi prep kit (Sigma-Aldrich, St. Louis, MO, USA) before being injected into mice.

### 2.2. Cell Lines and In Vitro Studies

In this study, we used the human Huh7, Hep3B, HLF, SNU449, and MHCC97-H HCC cell lines. The cell lines were purchased from the American Type Culture Collection (ATCC; Manassas, VA, USA) and the Japanese Collection of Research Bioresources Cell Bank (JCRB; Osaka, Japan). All cell lines, after additional validation using short tandem repeat technology (Genetica DNA Laboratories, Burlington, NC, USA), were grown in a 5% CO_2_ atmosphere at 37 °C, in a medium supplemented with 10% fetal bovine serum (FBS; Sigma-Aldrich, St. Louis, MO, USA) and penicillin/streptomycin (Sigma-Aldrich). For stable transfection of *HA-SKP2* in Huh7 and SNU449 cell lines, stable transfectants were selected with cloning cylinders after 3–4 weeks in a medium containing Geneticin (600 μg/mL; Thermo Fisher Scientific, Waltham, MA, USA). In addition, the FASN inhibitors C75 (final concentration 25 µM, corresponding to the drug’s IC50 in these cells; Sigma-Aldrich) was administered to Huh7 and SNU449 cells for 48 h. Cell proliferation was evaluated at the 48 h time point using the BrdU Cell Proliferation Assay Kit (Cell Signaling Technology, Danvers, MA, USA) according to the manufacturer’s protocol. Apoptosis was determined using the Cell Death Detection Elisa Plus Kit (Roche Molecular Biochemicals, Indianapolis, IN, USA), following the manufacturer’s instructions. Specifically, cells were subjected to 24 h serum starvation, and apoptosis was assessed at the 48 h time point. Cell cycle analysis was determined using 7AAD staining (BD Biosciences, Milpitas, CA, USA) and acquisition with a flow cytometer FACS CANTOII (BD Biosciences, CA, USA) following the manufacturer’s protocol.

### 2.3. Human Tissue Samples

Two hundred ten hepatocellular carcinoma samples were used for the study. These liver tumor specimens were archival tissues collected over the last 12 years at the Universities of Regensburg (Regensburg, Germany) and Greifswald (Greifswald, Germany). Only surgically resected specimens from patients who did not undergo prior treatments (chemotherapy, targeted therapies, etc.) for concurrent or previous primary tumors of the liver or other sites were included in the study. Tumors were divided into HCCs with shorter survival/poorer outcome (HCCP; n = 110) and longer survival/better outcome (HCCB; n = 100) survival, characterized by <3 and ≥3 years’ survival following partial liver resection, respectively. Patients’ clinicopathological features are summarized in Table 1. Liver tissues were collected at the Medical Universities of Greifswald (Greifswald, Germany) and Regensburg (Regensburg, Germany). Institutional Review Board approval was obtained at the local Ethical Committees of the Medical Universities of Greifswald (approval code: BB 67/10; 3 June 2010) and Regensburg (approval code: 17-1015-101; 4 July 2018), in compliance with the Helsinki Declaration. Written informed consent was obtained from all individuals.

### 2.4. Mouse Experiments

Some of the samples used in this investigation derive from previous studies conducted in our laboratory [21]. In particular, *Fasn^fl/fl^* conditional knockout mice (maintained on a C57BL/6J background) have been described previously in detail [21,40]. Hydrodynamic tail vein gene delivery was performed as reported exhaustively before. In brief, to study the requirement of FASN for AKT-driven hepatocarcinogenesis, six- to eight-week-old female *FASN^fl/fl^* mice were co-injected with myristoylated/activated AKT1 (myr-AKT1; 8 µg) and Cre recombinase (40 µg) plasmids (referred to as AKT/Cre mice; n = 5). This strategy allows the efficient Cre-dependent deletion of the *FASN* gene in the hepatocytes transfected with myr-AKT1. Additional *FASN^fl/fl^* mice were injected with myr-AKT1 (8 µg) and pT3 (40 µg) constructs as control (AKT mice; n = 5). To assess the relevance of SKP2 in AKT-induced HCC development, the myr-AKT1 (8 µg) and SKP2dn (40 µg) plasmids were co-injected into the liver of six- to eight-week-old female C57BL/6J mice (AKT/SKP2dn mice; n = 5). To determine the role of p27^KIP1^ as a critical effector of SKP2 in AKT-driven hepatocarcinogenesis, the myr-AKT1 (8 µg) and p27^KIP1-T187A-V5^ (40 µg) constructs were hydrodynamically administered to six- to eight-week-old female C57BL/6J mice (AKT/p27^KIP1^ mice). Specifically, p27^KIP1^T187A is a permanently active mutant form of p27^KIP1^ that cannot be phosphorylated and degraded by SKP2 [41,42].

Mice were maintained and monitored following the protocol approved by the Institutional Animal Care and Use Committee (IACUC, AN108577, 9 December 2004) of the University of California San Francisco (San Francisco, CA, USA).

### 2.5. Gene Knockdown and Transient Transfection

For gene silencing studies, human Huh7, Hep3B, HLF, MHCC97-H, and SNU449 cell lines were subjected to treatment with 30 nmol/L small interfering RNA (siRNA) targeting human *FASN* (# s5032; Thermo Fisher Scientific). We transfected the cell lines with Lipofectamine RNAiMAX (Thermo Fisher Scientific) following the manufacturer’s recommendations. A scrambled siRNA (# 4390846; Thermo Fisher Scientific) served as a negative control. For transient transfection experiments, 2 µg HA-Tagged SKP2, HA-tagged SKP2dn, or p27^KIP^T187A-V5-PT3EF5a constructs were injected in the HLF or SNU449 HCC cell lines using the Lipofectamine 2000 Reagent (Thermo Fisher Scientific) following the manufacturer’s protocol. Experiments were repeated at least three times in triplicate.

### 2.6. Quantitative Reverse Transcription Real-Time Polymerase Chain Reaction (qRT-PCR)

Gene Expression Assays for human *FASN* (Hs01005622_m1), *SKP2* (Hs01021864_m1), *CDKN1B* (Hs00153277_m1), and *β-actin* (4333762T), and mouse *Fasn* (Mm00662319_m1), *Skp2* (Mm00449925_m1), *Cdkn1b* (Mm00438168_m1) and *β-actin* (mm00607939_S1) were from Applied Biosystems (Foster City, CA, USA). Quantitative values for each gene were calculated using the PE Biosystems Analysis software and expressed as the number target (NT). NT = 2^−ΔCt^. We calculated each sample’s ΔCt value by subtracting the target gene’s average Ct value from the target gene’s average Ct value of the *β-Actin* gene. Experiments were repeated at least three times in triplicate.

### 2.7. Protein Extraction and Western Blot Analysis

Human cell lines and mouse liver tissues were homogenized in the M-PER Mammalian Protein Extraction Reagent (Thermo Fisher Scientific) containing the Complete Protease Inhibitor Cocktail (Roche Molecular Biochemicals, Indianapolis, IN, USA). Protein concentrations were determined using the Bio-Rad Protein Assay Kit (Bio-Rad, Hercules, CA, USA) and bovine serum albumin as standard. For Western blot analysis, aliquots of 40 μg were denatured by boiling in Tris-MOPS-SDS Running Buffer, separated by SurePAGE (Genscript, Piscataway, NJ, USA), and transferred onto nitrocellulose membranes (Bio-Rad) by electroblotting in Towbin buffer (25 mM Tris, 195 mM glycine, and 20% methanol). Membranes were blocked in Pierce Protein-free Tween 20 Blocking Buffer (Thermo Fisher Scientific) for 1 h and probed with the following specific antibodies: anti-FASN (# 3180; Cell Signaling Technology), SKP2 (# ab183039; Abcam, Cambridge, United Kingdom), p27 (# 3688; Cell Signaling Technology), and anti-β-Actin (# ab20272; Abcam). Each primary antibody was followed by incubation for 30 min with horseradish peroxidase secondary antibody (Jackson ImmunoResearch Laboratories, Inc., West Grove, PA, USA), diluted 1:5000. Anti-β-Actin (Sigma-Aldrich) antibody was used as a loading control. In addition, Ponceau S Red (Sigma-Aldrich) reversible staining confirmed equal loading and transfer of proteins onto the membranes. Finally, proteins were revealed with the Clarity Western ECL Substrate (Bio-Rad). Western blot analysis was repeated at least three times.

### 2.8. Histology and Immunohistochemistry

Mouse and human liver lesions, fixed in 4% paraformaldehyde overnight at 4 °C and embedded in paraffin, were evaluated by two board-certified pathologists and liver experts (M.E. and S.R.) following the criteria by Frith et al. [43]. For immunohistochemistry, antigen retrieval was achieved in 10 mM sodium citrate buffer (pH 6.0) by placing in a microwave on high for 10 min, followed by a 20 min cool down at room temperature. After a blocking step with the 5% goat serum and Avidin-Biotin blocking kit (Vector Laboratories, Burlingame, CA, USA), the slides were incubated with primary antibodies overnight at 4 °C. Slides were then subjected to 3% hydrogen peroxide for 10 min to quench endogenous peroxidase activity. Subsequently, the biotin-conjugated secondary antibody was applied at a 1:500 dilution for 30 min at room temperature. The anti-FASN (# 3180; 1:200 dilution; Cell Signaling Technology), anti-SKP2 (# ab183039; 1:50 dilution; Abcam), HA-Tag (# 3724; 1:400; Cell Signaling Technology), and V5-tag **(**# R960-25; 1:100; Thermo Fisher Scientific) antibodies were applied for immunohistochemistry. The immunohistochemical reaction was revealed with the Vectastain ABC-Elite Peroxidase Kit (# PK-6100; Vector Laboratories, Burlingame, CA, USA), using the ImmPACT NovaRed Substrate Peroxidase (# SK-4805; Vector Laboratories) as the chromogen. Slides were counterstained with Mayer’s hematoxylin.

### 2.9. Statistical Analysis

GraphPad Prism, version 9.0 (GraphPad Software Inc., La Jolla, CA, USA) was used to evaluate statistical significance by Tukey–Kramer and Student’s *t* tests. Patient survival was analyzed using Kaplan–Meier curves and the log-rank (Mantel–Cox) test. Variables were incorporated into the multivariate Cox model to identify the independent prognostic factors. Values of *p* < 0.05 were considered significant. Data are expressed as mean ± SD.

### 2.10. Graphical Work

Schemes and graphical representations were created using the BioRender.com online software (accessed on 13 May 2024).

## 3. Results

### 3.1. SKP2 Is a Downstream Effector of FASN in Hepatocellular Carcinoma Cell Lines

**Figure 1:**
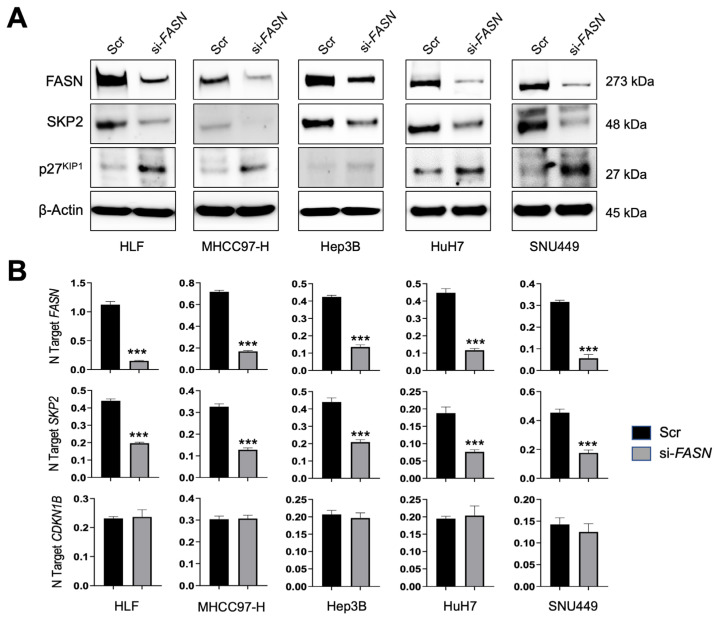
SKP2 is a FASN target in human HCC cell lines. (**A**) The HLF, MHCC97-H, Hep3B, HuH7, and SNU449 cell lines were subjected to *FASN* knockdown using a specific small interfering siRNA against *FASN* (si-FASN). Data were collected 48 h after the silencing. The effects of *FASN* silencing on FASN, SKP2, and p27^KIP1^ protein levels were detected by Western blot analysis. β-Actin was used as a loading control. (**B**) The effects of *FASN* silencing in the same cell lines on *FASN*, *SKP2*, and *CDKN1B* (encoding p27^KIP1^) mRNA levels were detected by quantitative real-time PCR. Student’s *t*-test: *p* < 0.0001 *** vs. scramble siRNA (Scr). Experiments were conducted three times in triplicate.

To determine whether SKP2 is a downstream target of FASN in liver cancer, five HCC cell lines (HLF, MHCC97-H, Hep3B, HuH7, and SNU449) were subjected to *FASN* gene silencing using a specific small interfering RNA (siRNA; Figure 1). As expected, FASN was effectively knocked down in all the cell lines tested. Of note, the silencing of FASN led to the downregulation of SKP2 in all the examined cells. The downregulation occurred at the mRNA and protein levels (Figure 1A,B).

To exert its oncogenic activity, mainly by inducing the cell cycle progression, SKP2 triggers the degradation of the p27^KIP1^ tumor suppressor, a negative cell cycle checkpoint [30,31,35]. Thus, we evaluated whether SKP2 downregulation following FASN silencing resulted in the induction of p27^KIP1^. As expected, suppression of the *SKP2* gene by *FASN* gene knockdown triggered p27^KIP1^ upregulation. Notably, the latter effect occurred at the protein (Figure 1A) but not the mRNA level (Figure 1B), consistent with the established post-transcriptional regulation of p27^KIP1^ by SKP2 [30,31,35]. Indeed, mRNA levels of *CDKN1B*, encoding the p27^KIP1^ cell cycle inhibitor, were not significantly different between scramble and SKP2-depleted cells.

**Figure 2:**
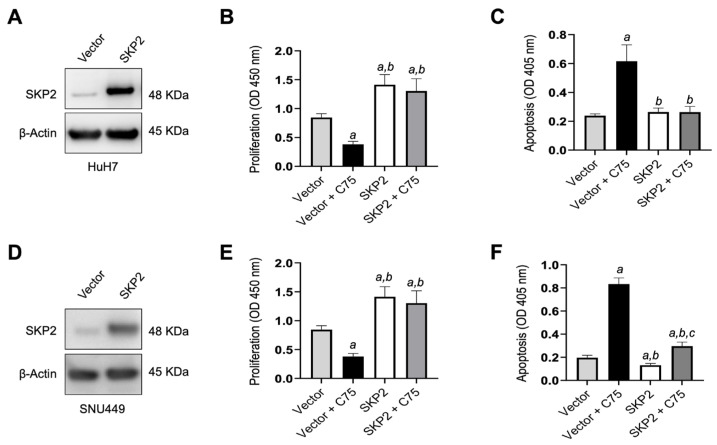
Forced overexpression of SKP2 confers resistance to HCC cells against growth restraint by the C75 FASN inhibitor. The Huh7 cell line was subjected to *SKP2* overexpression by stable transfection using an HA-tagged SKP2 plasmid (SKP2). Huh7 cells were also transfected with the empty vector (vector) as the control. Cells transfected with either *SKP2* or vector were grown untreated or treated with the FASN inhibitor C75 (25 µM) for 48 h. (**A**) Increased levels of SKP2 protein following transfection of the *SKP2* plasmid in Huh7 cells, as detected by Western blot analysis. β-Actin was used as a loading control. Transfection of *SKP2* increases proliferation (**B**) and diminishes apoptosis (**C**) in the same cells. C75 administration significantly reduces proliferation and augments apoptosis in vector-transfected but not SKP2-transfected cells. (**D**–**F**) Equivalent results were obtained in the SNU449 cell line. Tukey–Kramer’s test: *p* < 0.0001; *a*, versus empty vector (vector); *b*, versus vector-transfected and C75-treated cells (Vector + C75); *c,* versus SKP2 overexpression. Experiments were conducted three times in triplicate.

**Figure 3:**
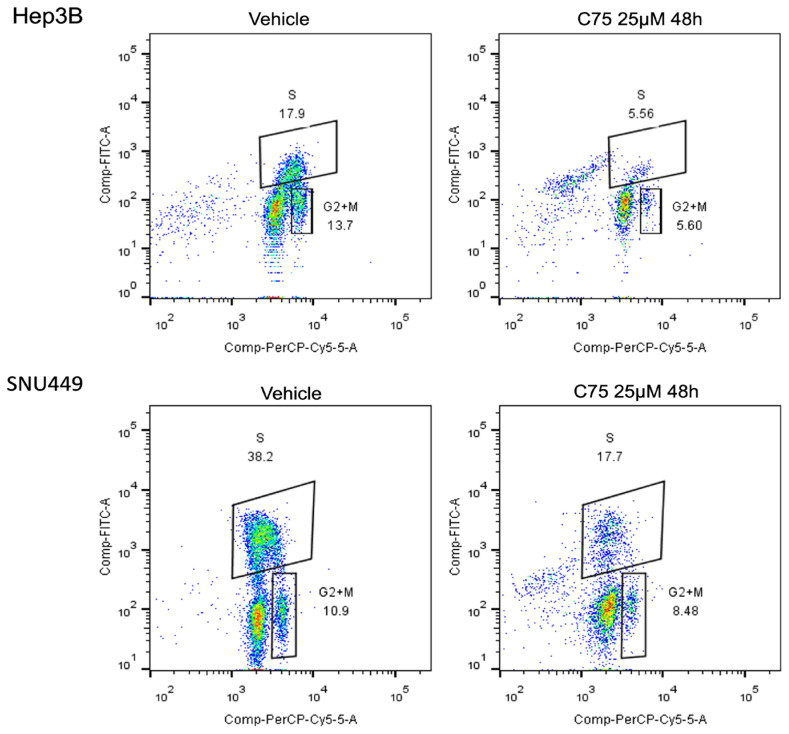
Dot spot graph representative of cell cycle distribution of HCC cells treated with C75 25 μM for 48 h. For the analysis, cells were recovered, washed with PBS and the pellet solubilized in ethanol 70% and stored at −20 °C overnight. The samples were stained with 7AAD (BD Biosciences, CA, USA) and incubated for 15 min at room temperature before acquisition using the flow cytometer FACS CANTOII (BD Biosciences, CA, USA). A total of 30.000 events for each sample were acquired and data were analyzed with ModFIT LT 6.0 (Verity Software House, Topsham, ME, USA).

**Figure 4:**
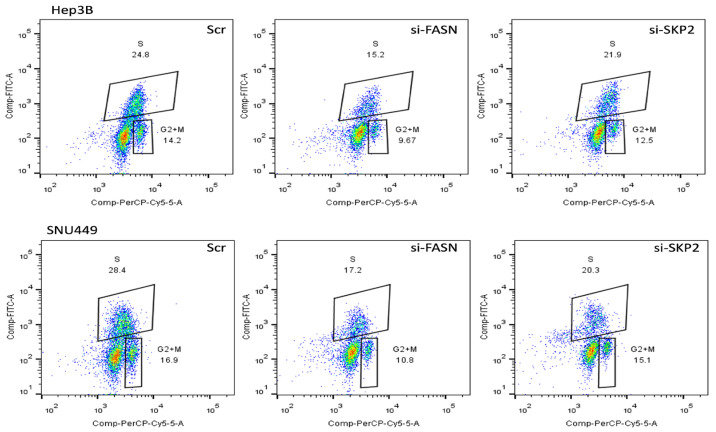
Dot spot graph representative of cell cycle distribution of HCC cells treated with si-FASN and si-SKP2 30 nM for 48 h. For the analysis, cells were recovered, washed with PBS and the pellet solubilized in ethanol 70% and stored at −20 °C overnight. The samples were stained with 7AAD (BD Biosciences, CA, USA) and incubated for 15 min at room temperature before acquisition using the flow cytometer FACS CANTOII (BD Biosciences, CA, USA). A total of 30.000 events for each sample were acquired and data were analyzed with ModFIT LT 6.0 (Verity Software House).

Subsequently, to further substantiate our findings, we determined whether forced overexpression of SKP2 in HuH7 cells might counteract the anti-growth effects of the FASN inhibitor C75 [44]. Therefore, the *SKP2* cDNA was stably expressed in Huh7 cells, and the empty vector was stably transfected as a control (Figure 2A). Administration of C75 induced significant growth restraint of Huh7 cells transfected with the empty vector compared to untreated cells by reducing proliferation (Figure 2B) and augmenting apoptosis (Figure 2C). In contrast, no growth differences were observed between untreated cells and those stably overexpressing *SKP2* treated with C75. SNU449 cells stably transfected with SKP2 displayed similar results (Figure 2D,E). Moreover, cell cycle analysis indicated a decrease in the S phase when HCC cells were subjected to C75 (25 µM) administration (Figure 3), si-FASN treatment, and, to a lesser extent, si-SKP2 treatment (Figure 4). C75 exerted a broader effect on HCC cells (Figure 3), presumably due to the possibility of affecting targets other than FASN, in accordance with previous findings [45]. These findings imply that the anti-growth properties of C75 are at least partly mediated by the drug’s effects on the FASN/SKP2 axis.

The present data indicate that SKP2 is a critical target of FASN in HCC cell lines.

### 3.2. Correlation of FASN and SKP2 Levels in Human Hepatocellular Carcinoma

**Figure 5:**
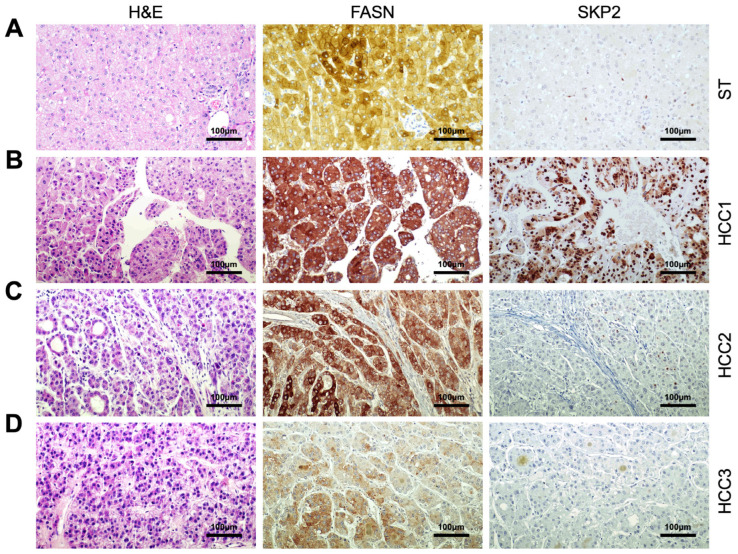
Representative immunohistochemistry patterns of FASN and SKP2 proteins in human hepatocellular carcinoma (HCC; n = 210). (**A**) Example of a liver non-tumorous surrounding tissue exhibiting moderate membranous and cytoplasmic for FASN and weak/absent immunolabeling for SKP2. (**B**) A human HCC (denominated HCC1) displaying robust and diffuse immunoreactivity for FASN and SKP2 proteins. Note that FASN staining is localized in the cytoplasm of HCC cells, whereas SKP2 immunoreactivity is localized in the cytoplasmic and nuclear compartments. (**C**) A second hepatocellular tumor (HCC2) is characterized by intense, homogeneous FASN immunoreactivity and low/absent SKP2 immunolabeling. (**D**) Finally, a third tumor (HCC3) shows faint FASN positivity and absent SKP2 immunoreactivity. Abbreviation: H&E, hematoxylin and eosin staining. Original magnifications: 200× in all panels. Scale bar: 100 µm in all panels.

Next, we determined whether FASN and SKP2 levels correlate in human HCC samples. For this purpose, we investigated the two proteins by immunohistochemistry in a large HCC collection (n = 210; Figure 5 and Supplementary Figure S1). Based on the patient’s survival, tumors were classified in HCC with shorter survival/poorer outcome (HCCP; n = 110) and longer survival/better outcome (HCCB; n = 100; Table 1). Increased FASN and SKP2 protein levels compared with corresponding non-tumorous liver tissues were detected in 158 and 130 samples, respectively (75.2% and 61.9%, respectively). Upregulation of FASN was more frequent in tumors with shorter survival (110/158, 69.6%). Similarly, most SKP2 upregulated samples belonged to the HCCP group (99/130, 76.1%). Furthermore, FASN immunoreactivity was localized in the cytoplasm of HCC cells, whereas malignant hepatocytes exhibited SKP2 immunolabeling in the cytoplasmic and/or nuclear compartments. The present data confirm our and other previous findings supporting the augmented SKP2 levels and nuclear localization mainly in the clinically aggressive HCC subset [38,46]. Immunohistochemical patterns did not correlate with other clinicopathological features of the patients, including gender, age, etiology, presence of cirrhosis, alfa-fetoprotein levels, tumor grade, and tumor stage.

**Figure 6:**
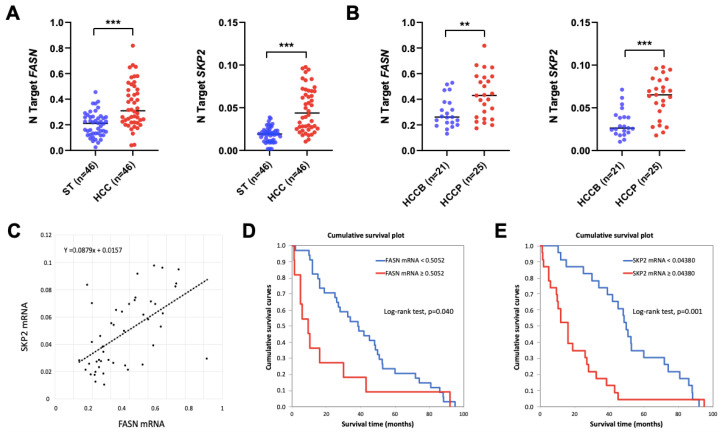
FASN and SKP2 are upregulated in human hepatocellular carcinoma (HCC). (**A**) Quantitative real-time RT-PCR values of *FASN* (first panel) and *SKP2* (second panel) are significantly higher in the tumors (HCC) (n = 46) than in corresponding non-tumorous counterparts (ST). (**B**) Quantitative real-time RT-PCR values of *FASN* (first panel) and *SKP2* (second panel) are significantly higher in HCC with poorer prognosis/shorter survival (HCCP; n = 25) than in tumors with better prognosis/longer survival (HCCB; n = 21). HCCP and HCCB are characterized by <3 and ≥3 years’ survival following partial liver resection. Student’s *t*-test: ***, *p* < 0.0001; **, *p* < 0.005. (**C**) Evaluation of the relation between *FASN* and *SKP2* mRNA in HCC samples. *p* values and correlation r values were calculated using Pearson correlation analysis. (**D**,**E**) Kaplan–Meyer curves in HCC patients show that *FASN* (**D**) and *SKP2* (**E**) mRNA levels inversely correlate with patients’ survival in this disease.

Subsequently, the mRNA levels of *FASN* and *SKP2* were investigated in the HCC samples from the same collection for which frozen tissues were available (n = 46; Figure 6). Levels of *FASN* and *SKP2* were significantly higher in HCC specimens compared with corresponding non-tumorous surrounding tissues (*p* = 2.42625 × 10^−6^ and *p* = 1.04229 × 10^−10^, respectively; Figure 6A). In addition, the expression of *FASN* and *SKP2* genes was more elevated in HCCP than in HCCB specimens (*p* = 0.004935 and *p* = 1.68832 × 10^−5^, respectively; Figure 6B). Furthermore, the mRNA levels of *FASN* and *SKP2* were directly correlated in the human HCC specimens analyzed, as detected via the Pearson correlation coefficient and Spearman correlation coefficient (non-parametric; *p* < 0.0001), as well as with a scatter plot (Figure 6C). These findings further substantiate the interaction between FASN and SKP2 in hepatocellular carcinogenesis. Subsequently, we determined whether the mRNA levels of *FASN* and *SKP2* were associated with patients’ survival. Patients’ specimens were stratified into low and high gene expression according to cut-points selected using a minimal *p*-value approach. Kaplan–Meier analysis revealed that a high expression of FASN and SKP2 is associated with poor prognosis (Figure 6D,E). Cox regression analysis confirmed that high levels of both FASN and SKP2 are independent adverse prognostic factors for HCC (Supplementary data).

### 3.3. Depletion of FASN Inhibits AKT-Driven Hepatocarcinogenesis and Downregulates SKP2 in Mice

**Figure 7:**
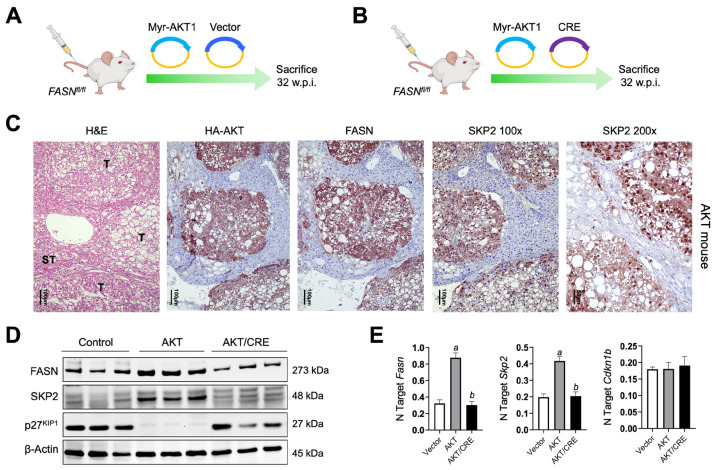
SKP2 is induced in the liver lesions from AKT mice. (**A**,**B**) Hydrodynamic gene delivery approach. In brief, *FASN^fl/fl^* mice were either injected with the myr-AKT1 construct (AKT mice) (**A**) or co-injected with Myr-AKT1 and Cre recombinase plasmids (AKT/Cre mice) (**B**). Five mice per group were injected and sacrificed 32 weeks post-injection (w.p.i.). (**C**) Immunohistochemical analysis shows that hepatocellular tumor lesions (T) developed in AKT mice display robust immunoreactivity for FASN, HA-AKT, and SKP2 proteins. Note that in the tumor cells, the immunoreactivity for SKP2 is localized in the cytoplasmic and nuclear compartments, as appreciable at the 200× magnification. In contrast, the surrounding non-tumorous liver tissues (ST) show weak/absent staining for the same proteins. Abbreviation: H&E, hematoxylin and eosin staining. Original magnifications: 100× and 200×, as indicated. Scale bar: 100 µm in 100× magnification pictures, 50 µm in the 200× magnification picture. (**D**) Representative Western blot analysis showing the levels of FASN, SKP2, and ^p27KIP1^ in livers from *FASN^fl/fl^* mice injected with the empty vector only (Control), Myr-AKT1 (AKT mice), and myr-AKT1/Cre (AKT/Cre mice). Note that AKT mice display upregulation of SKP2 and marked downregulation of p27^KIP1^. Suppression of FASN in AKT/Cre mice, which triggers the inhibition of hepatocarcinogenesis, is accompanied by downregulation of SKP2 and an increase of p27^KIP1^ protein levels. β-Actin was used as a loading control. (**E**) Quantitative real-time RT-PCR showing the mRNA levels of *Fasn*, *Skp2*, and *Cdkn1b* in livers from *FASN^fl/fl^* mice injected with the empty vector only (vector), Myr-AKT1 (AKT mice) and myr-AKT1/Cre (AKT/Cre mice). N target = 2^−ΔCt^, wherein the ΔCt value of each sample was calculated by subtracting the average Ct value of the target gene from the average Ct value of the β-*actin* gene. Five mice per group were analyzed. Tukey–Kramer’s test: *p* < 0.0001; *a*, versus control livers (vector); *b*, versus AKT livers.

Next, we determined the status of SKP2 in the hepatocellular lesions developed following the hydrodynamic injection of myr-AKT1 in *FASN^fl/fl^* mice harboring FASN (AKT mice; n = 5) or genetically deprived of this lipogenic protein (AKT/Cre mice; n = 5) (Figure 7A,B). These mice were previously developed [15,21], and Cre-mediated FASN deletion completely abolished AKT-dependent hepatocarcinogenesis in mice [21]. Immunohistochemistry detected homogeneous nuclear and cytoplasmic immunoreactivity for SKP2 in liver lesions developed in AKT mice, roughly co-localizing with the staining for HA-tagged AKT. In contrast, non-tumorous surrounding liver tissues displayed very low or absent SKP2 immunoreactivity (Figure 7C and Supplementary Figure S2, upper panels). As expected, HA-tagged AKT, FASN, and SKP2 immunoreactivity was negative in histologically normal livers from AKT/Cre mice (Supplementary Figure S2, lower panels). In addition, quantitative real-time RT-PCR and Western blot analyses confirmed SKP2 upregulation in AKT lesions, accompanied by downregulation of p27^KIP1^ protein levels in the same lesions (Figure 7D). Importantly, FASN suppression, which completely blunts AKT-dependent hepatocarcinogenesis [21], resulted in the downregulation of SKP2 and the upregulation of p27^KIP1^ protein in AKT mice depleted of FASN (AKT/Cre mice; Figure 7D). Again, like that observed in HCC cell lines, the downregulation of SKP2 occurred both at protein and mRNA levels. In contrast, p27^KIP1^ downregulation was detected only at the protein level (Figure 7D,E).

Overall, the present findings indicate that AKT-driven hepatocarcinogenesis is associated with SKP2 upregulation and the suppression of p27^KIP1^.

### 3.4. Inhibition of SKP2 Activity or Induction of p27^KIP1^ Abolishes AKT-Dependent Hepatocarcinogenesis

**Figure 8:**
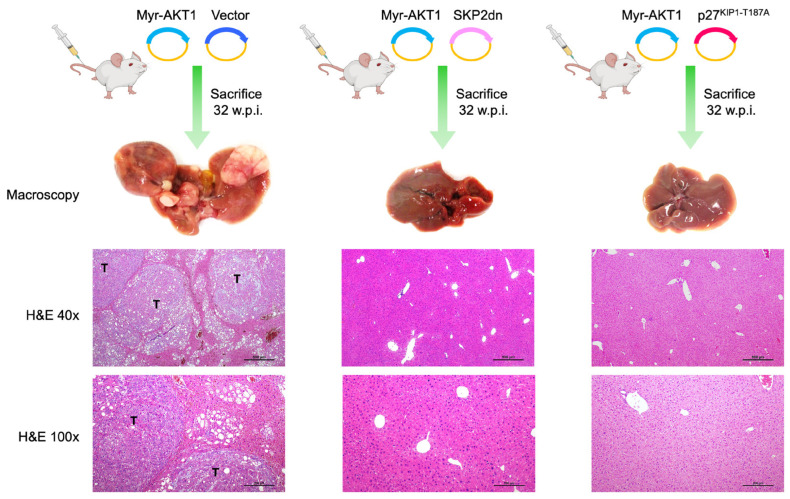
SKP2 inactivation or non-degradable p27^KIP1^ suppresses AKT-dependent hepatocarcinogenesis in mice. In the upper panels, the hydrodynamic gene delivery approach is depicted. In brief, C57BL/6J mice were either co-injected with the HA-tagged myr-AKT1 and empty vector (AKT mice), with Myr-AKT1 and SKP2 dominant negative (AKT/SKP2dn mice), or with Myr-AKT1 and a non-degradable form of V5-tagged p27^KIP1^ (p27^KIP1-T187A^; AKT/p27^KIP1^ mice). Five mice per group were injected and sacrificed 32 weeks post-injection (w.p.i.). At this time point, as revealed by hematoxylin and eosin staining (H&E), the livers of AKT mice are occupied by several tumor nodules (T). In contrast, the livers of AKT/SKP2dn and AKT/p27^KIP1^ mice appear completely normal (better appreciable in the pictures taken at higher magnification). Original magnifications: 40× and 100×. Scale bar: 500 µm in 40× magnification pictures, 200 µm in 100× magnification pictures.

Next, we sought to directly assess the relevance of SKP2 in AKT-driven mouse HCC development. For this reason, the myr-AKT1 plasmid was co-injected with the SKP2 dominant negative construct (*SKP2dn*) in the mouse liver via hydrodynamic gene delivery (n = 5; AKT/SKP2dn mice) (Figure 8). It has been previously shown that *SKP2dn* counteracts the activity of the *SKP2* wild-type gene, thus increasing p27^KIP1^ stability and half-life [30]. As a control, mice were injected with myr-AKT1 only (AKT mice; n = 5). Notably, the co-expression of *SKP2dn* with *myr-AKT1* inhibited AKT-driven liver carcinogenesis in mice. Indeed, the AKT/SKP2dn mice livers were completely normal 32 weeks post-injection, as assessed by histopathologic analysis. In contrast, all mice injected with myr-AKT1 displayed multiple tumors on the liver surface, confirmed at the microscopical level. Subsequently, we investigated whether SKP2 favors AKT-dependent HCC development through its ability to downregulate p27^KIP1^. Thus, we hydrodynamically injected myr-AKT1 in association with p27^KIP1-T187A^, a mutant form of p27^KIP1^ that SKP2 cannot downregulate [41,42]. Notably, co-expression of *myr-AKT1* with *p27^KIP1-T187A^* (n = 5; AKT/p27^KIP1^ mice) resulted in the complete abolishment of AKT-dependent hepatocarcinogenesis in AKT/p27^KIP1^ mice. Like AKT/SKP2dn mice, the livers of AKT/p27^KIP1^ mice showed no liver histopathologic alterations (Figure 8). Four additional AKT/p27^KIP1^ mice were sacrificed 5 weeks post hydrodynamic injection (Supplementary Figure S3). Interestingly, at this time, few enlarged lipid-rich hepatocytes were observed in the liver parenchyma of AKT/p27KIP1 mice. In particular, these cells displayed positive immunoreactivity for HA-AKT and V5-p27^KIP1-T187A^ tags, implying their origin from the injected genes. The lack of these cells at 32 weeks post-injection suggests their elimination during AKT/p27KIP1 mouse aging.

The in vivo data indicate that SKP2 is a critical contributor to AKT-dependent hepatocarcinogenesis downstream of FASN via its ability to downregulate the p27^KIP1^ tumor suppressor.

### 3.5. Induction of p27^KIP1^ by SKP2dn or p27^KIP1^ Overexpression Reduces Proliferation and Increases Apoptosis in HCC Cells In Vitro

**Figure 9:**
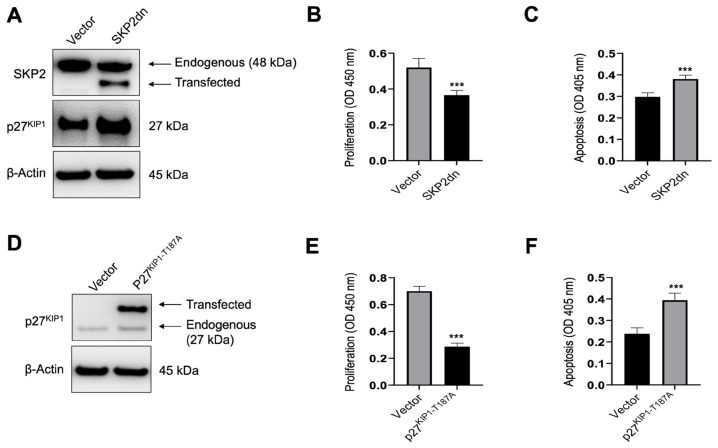
Inactivation of SKP2 or non-degradable p27^KIP1^ is detrimental to the growth of HCC cells in vitro. (**A**) Transfection of *SKP2dn* triggers the upregulation of p27^KIP1^ levels in SNU449 cells, as detected by Western blot analysis. As expected, transient transfection of *SKP2dn* resulted in the expression of a truncated form of SKP2 (transfected) with a lower molecular weight than the endogenous protein. β-Actin was used as a loading control. Transfection of SKP2dn reduces proliferation (**B**) and increases apoptosis (**C**) in the same cells. (**D**) Transfection of *p27^KIP1−187A^* results in the appearance of a second band (transfected) with a higher molecular weight than the endogenous p27^KIP1^ protein in the SNU449 cell line. β-Actin was used as a loading control. Similar to that described for SKP2dn, transfection of *p27^KIP1−187A^* decreases proliferation (**E**) and augments apoptosis (**F**) in the same cell line. Student’s *t*-test: *p* < 0.0001 *** vs. empty vector (control). Experiments were conducted three times in triplicate.

Finally, we assessed the effect of *SKP2dn* and *p27^KIP1-T187A^* overexpression on the in vitro growth of HCC cell lines. For this purpose, the SNU449 HCC cell line was subjected to *SKP2dn* and *p27^KIP1-T187A^* transient transfection. The effects of the forced overexpression of the two genes on the proliferation and apoptosis of SNU449 cells were evaluated 48 h after transfection (Figure 9). We found that overexpression of either *SKP2dn* or *p27^KIP1-T187A^* resulted in the decline in proliferation and induction of apoptosis (Figure 9A–F). Thus, the reactivation of *p27^KIP1^* is detrimental to the growth of HCC cells in vitro.

## 4. Discussion

Aberrant expression and activity of FASN is a hallmark of cancer development and progression in several tumor types, including human HCC [10,11,12,13,14,15,16]. Consequently, FASN inhibitors have been tested as anticancer agents in numerous preclinical in vitro and in vivo models [19,47,48]. In addition, the FASN inhibitor TVB-2640 [49] is currently being evaluated in various clinical trials involving multiple solid cancers. Specifically, TVB-2640 studies investigate its potential use in monotherapy and combination treatments against various solid tumors (https://clinicaltrials.gov (accessed on 13 May 2024); NCT03808558, NCT02223247, NCT04352361, NCT03938246, NCT02980029, NCT04906421, NCT03032490411, and NCT0317917517). Even though these trials are still in the recruiting phase, preliminary findings show good tolerability and an improvement in antineoplastic activity when combined with other drugs against non-small cell lung cancer (NCT03808558) and breast cancer (NCT03179904). Nonetheless, a recent study indicates that FASN inhibition is highly detrimental to the growth of prostate carcinoma cell lines, while it does not affect the oncogenic properties of pancreatic adenocarcinoma cells [50]. Similarly, we previously revealed that the genetic deletion of the *Fasn* gene suppresses carcinogenesis in mouse models of HCC [20,21] but is ineffective against murine intrahepatic cholangiocarcinoma development [51]. Therefore, FASN inhibition is presumably helpful against some but not all tumor entities, and reliable biomarkers are needed to discriminate tumors based on their sensitivity to FASN inhibitors. Furthermore, the mechanisms related to FASN-dependent pro-oncogenic activity should be better delineated to understand how FASN supports carcinogenesis and the possible vulnerabilities of FASN-dependent tumors. A wealth of evidence indicates that FASN contributes to the malignant phenotype by inducing the synthesis of fatty acids necessary for the plasma membrane production of the cancer cells and lipid-based post-transcriptional protein modifications [13,52,53]. However, emerging data from high-throughput approaches reveal that FASN regulates many downstream effectors with different roles on cell behavior [26,29], suggesting that FASN also supports carcinogenesis via mechanisms beyond its lipogenic activity.

In the present investigation, we determined the functional importance of SKP2, a central component of the SCF-SKP2 ubiquitin ligase complex [33,54], as a potential FASN target in human HCC. Using liver cancer cell lines, mouse models, and human specimens, we found that SKP2 is a critical effector of FASN in this aggressive tumor. In particular, we showed that SKP2 inactivation phenocopies FASN deletion and abolishes murine hepatocarcinogenesis induced by AKT overexpression. Furthermore, we identified the p27^KIP1^ oncosuppressor and cell cycle regulator as the SKP2 critical target whose downregulation leads to AKT-driven HCC development in the mouse. Indeed, the co-expression of an unphosphorylatable/non-degradable form of p27^KIP1^ triggered the inhibition of hepatocarcinogenesis in AKT-injected mice. Notably, preliminary data from our laboratory show that *FASN* knockdown does not trigger the downregulation of SKP2 and the upregulation of p27^KIP1^ in colorectal cancer and glioblastoma cell lines (Cigliano et al., unpublished observation), suggesting that the existence of a FASN/SKP2/p27^KIP1^ axis might be a tumor-specific feature. Further studies are necessary to address this intriguing issue.

The mechanisms whereby FASN induces SKP2 remain undefined and are beyond the scope of the present study. Nonetheless, previous observations imply that FASN-mediated inhibition of the retinoblastoma (pRB) tumor suppressor pathway is the primary mechanism responsible for SKP2 induction in breast cancer cells [27]. On the other hand, our preliminary experiments seem to exclude such a mechanism in HCC cells (Cigliano et al., unpublished observation), indicating that the molecular events downstream of FASN triggering SKP2 upregulation might be multiple and context-dependent. In addition, suppression of stearoyl-CoA desaturase, a FASN downstream effector in the lipogenesis pathway, provided inconsistent results in terms of SKP2 levels (Cigliano et al., unpublished observation), questioning the possibility that FASN regulates SKP2 through its lipogenic activity. Nonetheless, we cannot exclude the fact that FASN controls SKP2 expression via indirect mechanisms. For instance, FASN might regulate SKP2 expression via microRNA modulation. Indeed, recent studies demonstrated that fatty acids are relevant regulators of microRNAs in the liver [55,56]. Therefore, it would be critical to investigate whether FASN regulates the expression of microRNAs in this organ. Furthermore, emerging evidence points to the role of FASN in driving protein translation [25]; thus, SKP2 might be one of the targets of this FASN activity.

Taken together, the data from this investigation strongly suggest that the inhibition of SKP2 or the reactivation of p27^KIP1^ might represent a valuable therapeutic option against HCC lesions characterized by the activation of the FASN cascade. These findings might have important clinical implications regarding innovative and targeted therapies against this deadly tumor. In this regard, numerous SKP2 inhibitors have been developed, although they have not yet reached clinical testing [33,57]. Likewise, no established p27^KIP1^ activators have been approved for treating cancer patients. Interestingly, the recently developed liposomal:peptide drug NP-ALT has shown the ability to suppress the tyrosine phosphorylation-mediated inhibition of p27^KIP1^ and to induce necroptosis and tumor regression in xenograft models of breast cancer [58], pointing to p27^KIP1^ as an effective anticancer target. Further studies will address the relevance of these therapeutic strategies for treating human HCC.

## 5. Conclusions

In summary, we revealed that SKP2 is a downstream effector of FASN in mouse hepatocarcinogenesis induced by overexpression of the AKT oncogene. In this model, suppressing SKP2 or reactivating p27KIP1, an SKP2 target, is sufficient to inhibit liver tumor development. In human HCC, FASN and SKP2 levels directly correlate and are associated with poor patient prognosis. Thus, targeting SKP2 or p27KIP1 might be an innovative therapeutic strategy against liver tumors with elevated FASN.

## Figures and Tables

**Table 1:** Clinicopathological features of HCC patients.

Variables	Features	
	HCCB	HCCP
Number of patients		
Male	15	17
Female	6	8
Age (Mean ± SD)	66.7 ± 7.46	67.2 ± 10.5
Etiology		
HBV	8	10
HCV	7	8
Ethanol	2	3
N/A	4	4
Cirrhosis		
+	14	18
−	7	7
Tumor size		
>5 cm	12	19
<5 cm	9	6
Edmondson and Steiner grade		
II	8	6
III	10	10
IV	3	9
Serum alpha-fetoprotein level (ng/mL)		
>300	14	19
<300	7	6
Survival after partial liver resection months		
(Mean ± SD)	61.5 ± 19.8	15.2 ± 10.0

Abbreviations: N/A: Not applicable, SD: Standard deviation.

## Data Availability

Data are contained within the article and Supplementary Materials. The data will be available on request.

## Supplementary Materials

The following supporting information can be downloaded at: https://www.mdpi.com/article/10.3390/medicina60071160/s1, Figure S1: Representative immunohistochemistry patterns of FASN and SKP2 proteins in human hepatocellular carcinoma specimens; Figure S2: Immunohistochemical analysis of AKT and AKT/CRE mouse livers; Figure S3: Overexpression of a non-degradable p27^KIP1^ form suppresses AKT-dependent hepatocarcinogenesis in mice.

## Abbreviations

FASN, fatty acid synthase; HCC, hepatocellular carcinoma; siRNA, small interfering RNA; SKP2, S-phase kinase-associated protein 2.
