# Supplementary material for: Fatty Acid Synthase Promotes Hepatocellular Carcinoma Growth via S-Phase Kinase-Associated Protein 2/p27KIP1 Regulation
Source: Medicina (Kaunas). 2024 Jul 18;60(7):1160. doi: 10.3390/medicina60071160 (PMC11278665; doi:10.3390/medicina60071160)
Supplement: Supplementary file 1 [file medicina-60-01160-s001.zip › Suppl. Fig. S1.pdf]

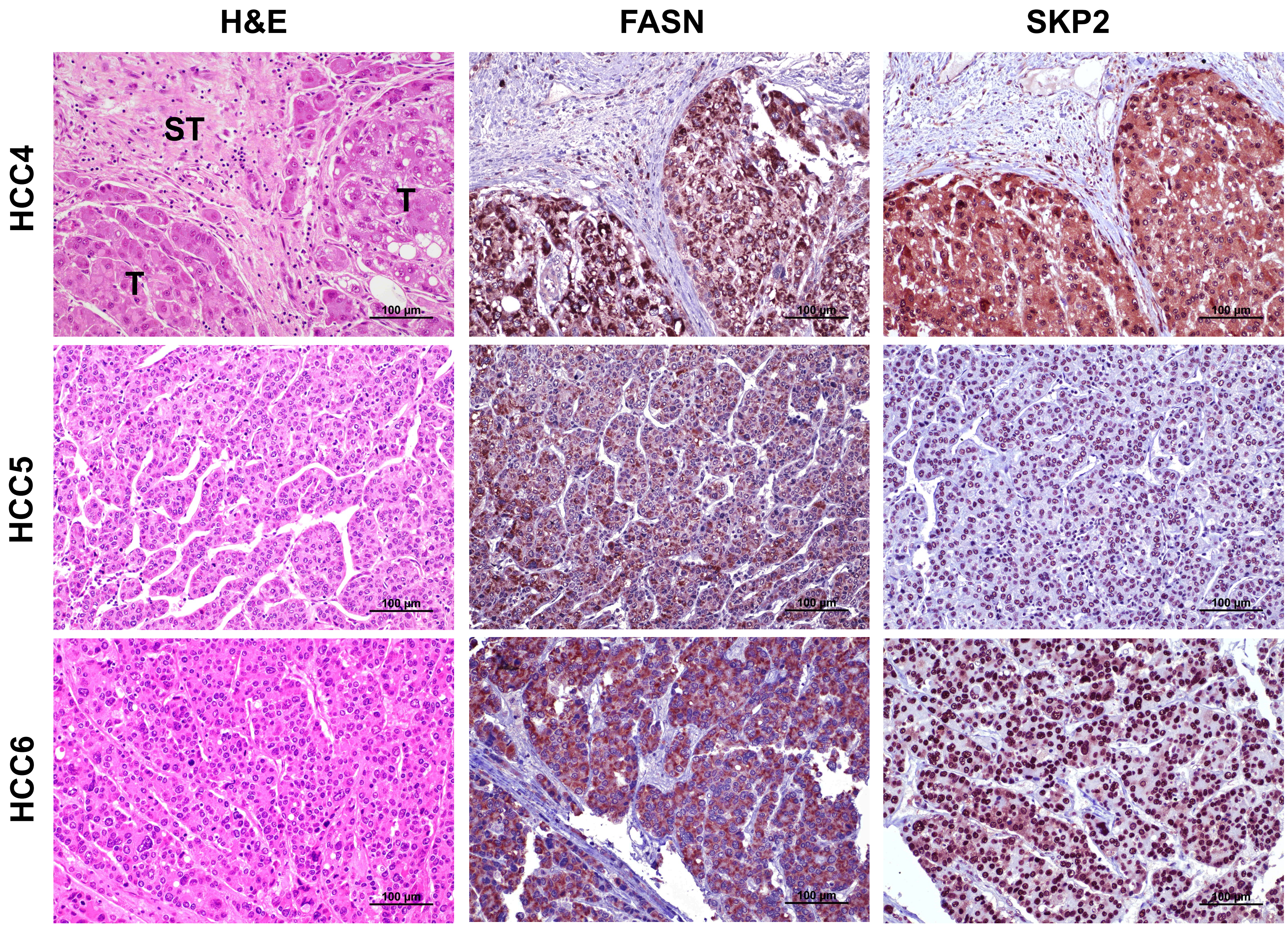
H&E
FASN
SKP2
HCC4
HCC5
HCC6
ST
T
T
100 μm
100 μm
100 μm
100 μm
100 μm
100 μm
100 μm
100 μm
100 μm

Supplementary Figure S1. Representative immunohistochemistry patterns of FASN and SKP2 proteins in human hepatocellular carcinoma specimens. Here, three HCC specimens (HCC4, HCC5, and HCC6) different from the ones depicted in Figure 5. The three tumors show intense membranous and cytoplasmic staining for FASN and nuclear and/or cytoplasmic immunolabeling for SKP2. In HCC4, the tumor part (T) displays more pronounced immunoreactivity for FASN and SKP2 compared with the adjacent surrounding tissue (ST). Abbreviation: H&E, hematoxylin and eosin staining. Original magnifications: 200x in all panels. Scale bar: 100 μm in all panels.
