# Supplementary material for: Fatty Acid Synthase Promotes Hepatocellular Carcinoma Growth via S-Phase Kinase-Associated Protein 2/p27KIP1 Regulation
Source: Medicina (Kaunas). 2024 Jul 18;60(7):1160. doi: 10.3390/medicina60071160 (PMC11278665; doi:10.3390/medicina60071160)
Supplement: Supplementary file 1 [file medicina-60-01160-s001.zip › Suppl. Fig. S2.pdf]

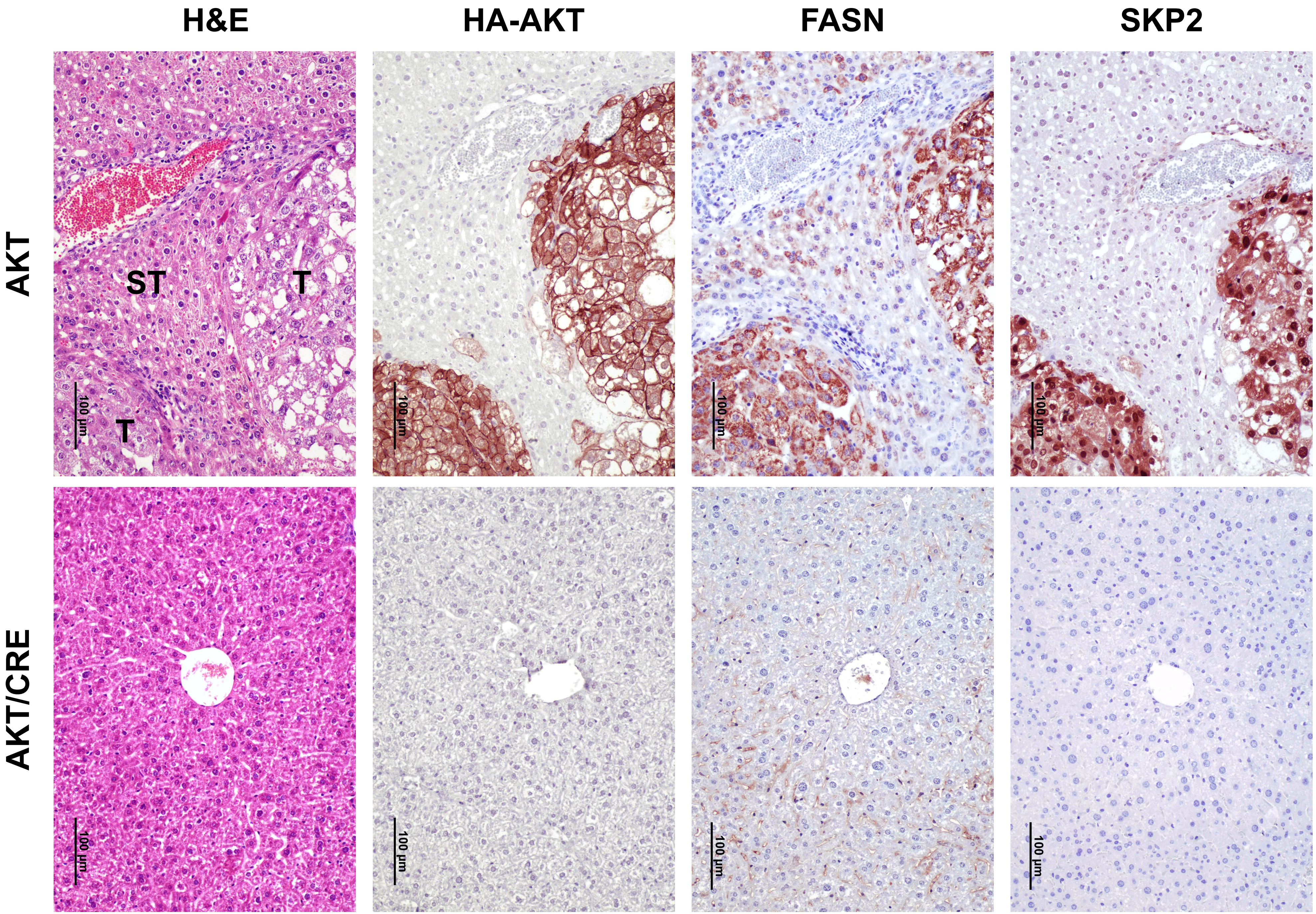
H&E
HA-AKT
FASN
SKP2
AKT
AKT/CRE
ST
T
T
100 µm

Supplementary Figure S2. Immunohistochemical analysis of AKT and AKT/CRE mouse livers. In the upper panels, hepatocellular tumor lesions (T) with a clear cell phenotype display pronounced immunoreactivity for HA-AKT, FASN, and SKP2 proteins compared with surrounding non-tumorous liver (ST). Immunolabeling is membranous and cytoplasmic for HA-AKT and FASN, whereas it is cytoplasmic and nuclear for SKP2. Lower panels: CRE-mediated deletion of FASN completely blunts hepatocarcinogenesis in AKT/CRE mice. Livers appear healthy and morphologically normal and exhibit weak or absent staining for HA-AKT, FASN, and SKP2 proteins. Abbreviation: H&E, hematoxylin and eosin staining. Original magnifications: 200x in all panels. Scale bar: 100 μm in all panels.
