# Supplementary material for: Fatty Acid Synthase Promotes Hepatocellular Carcinoma Growth via S-Phase Kinase-Associated Protein 2/p27KIP1 Regulation
Source: Medicina (Kaunas). 2024 Jul 18;60(7):1160. doi: 10.3390/medicina60071160 (PMC11278665; doi:10.3390/medicina60071160)
Supplement: Supplementary file 1 [file medicina-60-01160-s001.zip › Suppl. Fig. S3.pdf]

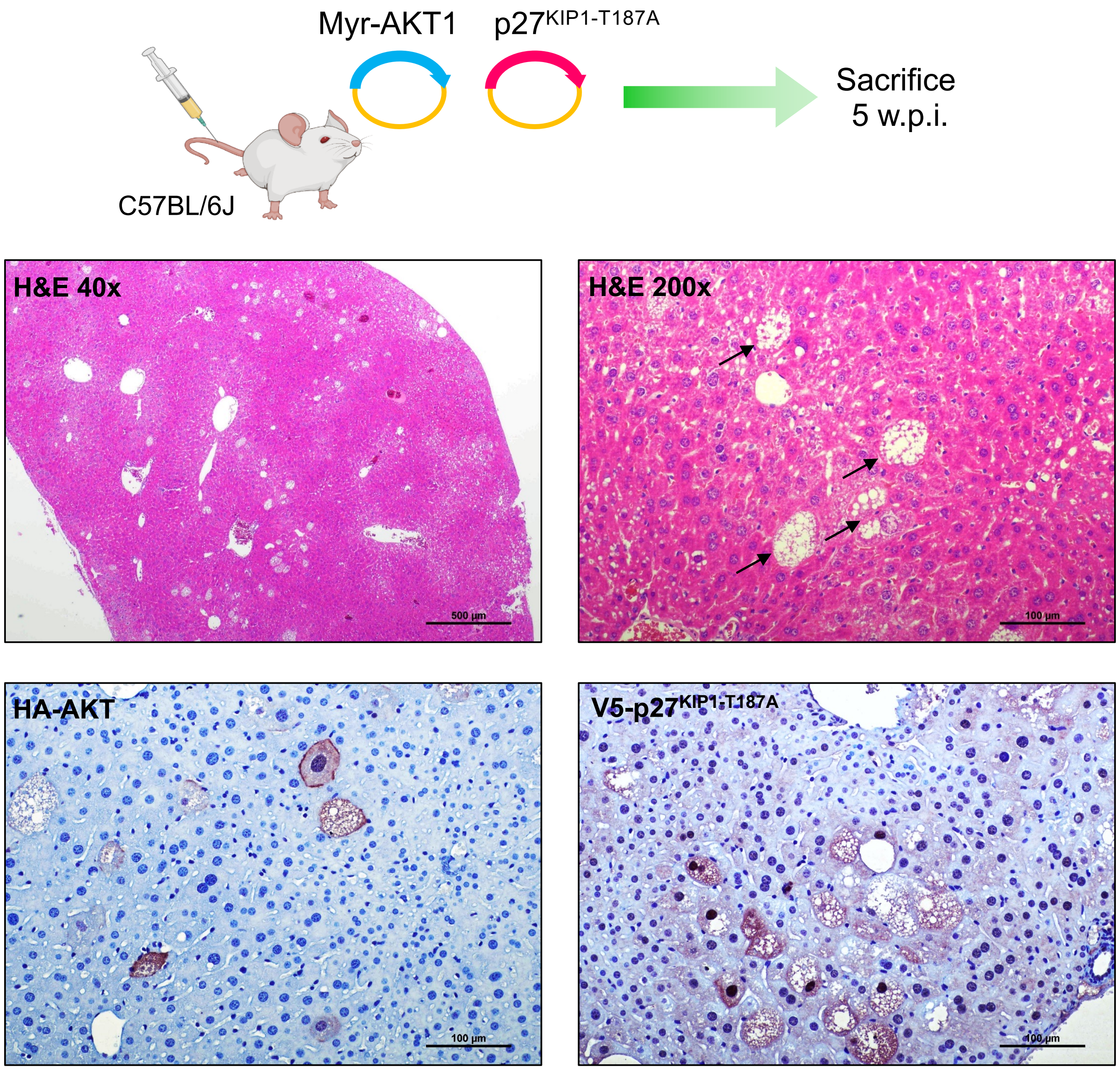

Myr-AKT1
p27KIP1-T187A
Sacrifice
5 w.p.i.
C57BL/6J
H&E 40x
500 µm
H&E 200x
100 µm
HA-AKT
100 µm
V5-p27KIP1-T187A
100 µm

Supplementary Figure S3. Overexpression of a non-degradable p27^{KIP1} form suppresses AKT-dependent hepatocarcinogenesis in mice. In the upper panel, the hydrodynamic gene delivery approach is depicted. In brief, four C57BL/6J mice were co-injected with the HA-tagged myr-AKT1 and a non-degradable form of V5-tagged p27^{KIP1} (p27$^{KIP1-T187A}$; AKT/p27^{KIP1} mice) and sacrificed 5 weeks post-injection (w.p.i.). At this time point, as revealed by hematoxylin and eosin staining (H&E) in the middle panels, the livers of AKT/p27^{KIP1} mice show the presence of few, scattered lipid-rich hepatocytes (indicated by arrows), within the normal liver parenchyma (better appreciable in the picture taken at higher magnification). These enlarged hepatocytes are immunoreactive for HA-AKT and V5-p27$^{KIP1-T187A}$ antibodies, implying their origin from the injected constructs. Original magnifications: 40x and 100x. Scale bar: 500 μm in 40x magnification pictures, 200 μm in 100x magnification pictures.
