# Supplementary material for: Fatty Acid Synthase Promotes Hepatocellular Carcinoma Growth via S-Phase Kinase-Associated Protein 2/p27KIP1 Regulation
Source: Medicina (Kaunas). 2024 Jul 18;60(7):1160. doi: 10.3390/medicina60071160 (PMC11278665; doi:10.3390/medicina60071160)

SUPPLEMENTARY MATERIAL

The analysis included data from 46 patients with human hepatocellular carcinoma (all with survival data). The variables have been analyzed using the Statistical Package for Social Science (SPSS, version 22.0, Chicago, IL, USA).

Descriptive statistics

**Sex**	**Number (%)**	**Mean age (SD)**	**Mean survival (SD)**
Male	32 (69.6)	67.47 (9.53)	36.66 (27.84)
Female	14 (30.4)	65.86 (8.48)	35.68 (28.49)
Total	46	66.98 (9.16)	36.36 (27.72)

Mean survival is 36.36 months (SD 27.72)
(differences are not statistically significant)

**Sex**	**Number (%)**	**Mean FASN mRNA (SD)**	**Mean SKP2 mRNA (SD)**
Male	32 (69.6)	0. 36859 (0.15216)	0.04928 (0.02654)
Female	14 (30.4)	0. 37069 (0.19530)	0.04545 (0.02498)
Total	46	0. 36923 (0.16422)	0.04812 (0.02586)

(differences are not statistically significant)

FASN **mRNA in human hepatocellular carcinoma**

Low and high *FASN mRNA* values were recoded into binary variables (0/1) using the median values (FASN mRNA = 0.32314) as the cut-off. The whole dataset was then divided into 23 subjects with values of FASN mRNA below the median, and 23 subjects above the median. Statistical comparison between the two groups was performed using the **log-rank test**:

**Marker**	**Number of subjects (%)**	**Mean survival in months (SD)**	**Log-rank test**
FASN mRNA < 0.32314	23 (50.0)	45.70 (25.36)	–
FASN mRNA ≥ 0.32314	23 (50.0)	27.01 (27.32)	0.119
Total	46	36.36 (27.72)	

Conclusion 1: *patients with FASN mRNA values above the median 0.32314* ***have a non-significant shorter survival than*** *patients with FASN mRNA below 0.32314 (see Kaplan-Meier curve).*

SKP2 **mRNA in human hepatocellular carcinoma**

Low and high *SKP2 mRNA* values were recoded into binary variables (0/1) using the median values (SKP2 mRNA = 0.04380) as the cut-off. The whole dataset was then divided into 23 subjects with values of SKP2 mRNA below the median, and 23 subjects above the median. Statistical comparison between the two groups was performed using the **log-rank test**:

**Marker**	**Number of subjects (%)**	**Mean survival in months (SD)**	**Log-rank test**
SKP2 mRNA < 0.04380	23 (50.0)	52.02 (24.99)	–
SKP2 mRNA ≥ 0.04380	23 (50.0)	20.70 (20.85)	0.001
Total	46	36.36 (27.72)	

Conclusion 2: *patients with SKP2 mRNA values above the median 0.04380* ***have shorter survival than*** *patients with SKP2 mRNA below 0.04380 (see Kaplan-Meier curve).*

Kaplan-Meier curves

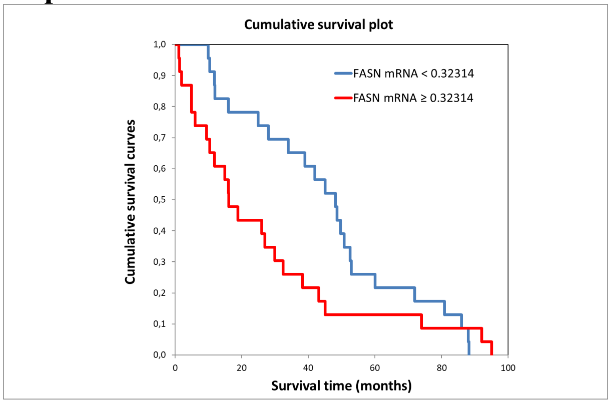

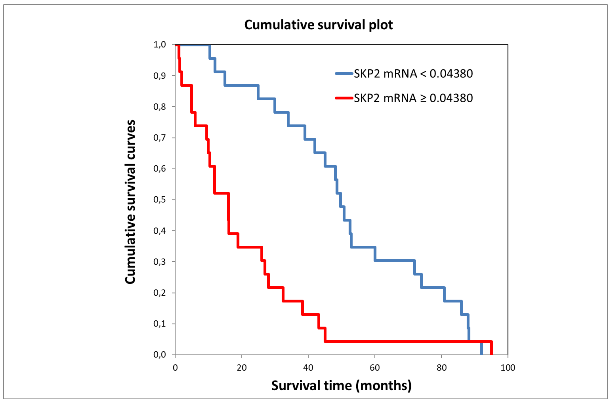

Survival analysis (univariate)

Variable	Survival (months) (SD)	p-value
Age (years)		
< 65	35.40 (27.68)	0.853
≥ 65	36.97 (28.24)	
Sex		
Male	36.66 (27.84)	0.914
Female	35.68 (28.49)	
Cirrhosis		
No	38.68 (24.10)	0.712
Yes	35.34 (29.47)	
Etiology		
HBV	32.86 (28.69)	0.379
HCV	34.26 (19.67)	
Ethanol	30.30 (31.99)	
Size		
< 3 cm	41.88 (26.37)	0.402
≥ 3 cm	34.18 (28.33)	
AFP		
< 300 ng/ml	43.42 (33.45)	0.283
≥ 300 ng/ml	33.58 (25.16)	
Grade		
II	42.89 (24.76)	0.122
III	40.17 (32.68)	
IV	22.39 (16.78)	
FASN mRNA		
< 0.32204	45.70 (25.36)	**0.020**
≥ 0.32204	27.01 (27.32)	
SKP2 mRNA		
< 0.04562	52.02 (24.99)	**<0.0001**
≥ 0.04562	20.70 (20.85)	

Multivariate Cox regression analysis

Two multivariate Cox proportional hazard models were created with survival as the outcome variable. FASN mRNA and SKP2 mRNA were included as predictors together with the covariates (full model). Hazard ratios (HRs) and their 95% confidence intervals were calculated, and the Wald test was used for model testing.

**Covariates**	**Full model<br>(HR and 95% CI)**	**Full model<br>(HR and 95% CI)**
Age ≥ 65 years	1.463 (0.587–3.645)	0.871 (0.342–2.214)
Male sex	0.991 (0.472–2.081)	1.122 (0.535–2.354)
Cirrhosis (y/n)	1.429 (0.674–3.031)	0.752 (0.365–1.552)
Etiology<br>HBV<br>HCV<br>Ethanol	 Reference<br>1.069 (0.465–2.459)<br>1.809 (0.587–5.574)	 Reference<br>0.476 (0.193–1.173)<br>1.582 (0.494–5.064)
Diameter ≥ 3 cm	1.075 (0.499–2.316)	1.301 (0.580–2.917)
AFP<br>< 300 ng/ml<br>≥ 300 ng/ml	 Reference<br>2.299 (0.889–5.949)	 Reference<br>3.163 (1.233–8.116) *
Grade<br>II<br>III<br>IV	 Reference<br>0.662 (0.247–1.776)<br>1.572 (0.639–3.867)	 Reference<br>0.967 (0.339–2.756)<br>0.963 (0.368–2.520)
FASN mRNA ≥ 0.32314 (median value)	2.518 (1.190–5.329) *	–
SKP2 mRNA ≥ 0.04380 (median value)	–	7.108 (2.869–17.61) **

*p<0.01; **p<0.001

Conclusion: ***FASN mRNA and SKP2 levels above the median were significantly associated with reduced survival.***

Correlation analysis

The correlation between FASN mRNA and SKP2 mRNA was carried out using the Pearson correlation coefficient and Spearman correlation coefficient (non-parametric), as well as with a scatter plot.

Pearson correlation coefficient (r)	0.558	P<0.0001
Spearman correlation coefficient (ρ)	0.576	P<0.0001

The correlation between FASN and SKP2 is highly significant (see scatterplot).

Scatterplot

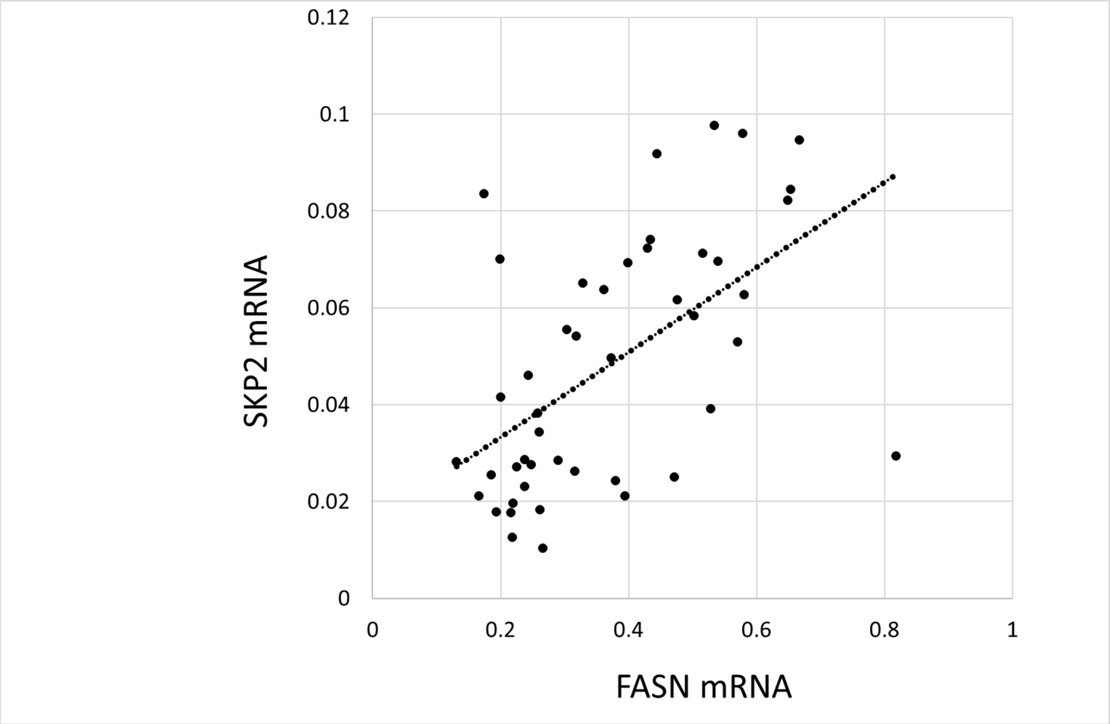

Supplement: Supplementary file 1 [file medicina-60-01160-s001.zip › Supplementary data.pdf]
